# Skin α-Synuclein Aggregation Seeding Activity as a Novel Biomarker for Parkinson Disease

**DOI:** 10.1001/jamaneurol.2020.3311

**Published:** 2020-09-28

**Authors:** Zerui Wang, Katelyn Becker, Vincenzo Donadio, Sandra Siedlak, Jue Yuan, Masih Rezaee, Alex Incensi, Anastasia Kuzkina, Christina D. Orrú, Curtis Tatsuoka, Rocco Liguori, Steven A. Gunzler, Byron Caughey, Maria E. Jimenez-Capdeville, Xiongwei Zhu, Kathrin Doppler, Li Cui, Shu G. Chen, Jiyan Ma, Wen-Quan Zou

**Affiliations:** 1 Department of Pathology, Case Western Reserve University School of Medicine, Cleveland, Ohio; 2 Center for Neurodegenerative Science, Van Andel Institute, Grand Rapids, Michigan; 3 IRCCS Institute of Neurological Sciences of Bologna, Complex Operational Unit Clinica Neurologica, Bologna, Italy; 4 Department of Neurology, University Hospital of Würzburg, Würzburg, Germany; 5 Laboratory of Persistent Viral Diseases, Rocky Mountain Laboratories, Hamilton, Montana; 6 Department of Neurology, University Hospitals Cleveland Medical Center, Case Western Reserve University School of Medicine, Cleveland, Ohio; 7 Faculty of Medicine, University of San Luis Potosi, San Luis Potosi, Mexico; 8 Department of Neurology, The First Hospital of Jilin University, Changchun, China; 9 National Center for Regenerative Medicine, Case Western Reserve University School of Medicine, Cleveland, Ohio

## Abstract

**Importance:**

Deposition of the pathological α-synuclein (αSyn^P^) in the brain is the hallmark of synucleinopathies, including Parkinson disease (PD), Lewy body dementia (LBD), and multiple system atrophy (MSA). Whether real-time quaking-induced conversion (RT-QuIC) and protein misfolding cyclic amplification (PMCA) assays can sensitively detect skin biomarkers for PD and non-PD synucleinopathies remains unknown.

**Objective:**

To develop sensitive and specific skin biomarkers for antemortem diagnosis of PD and other synucleinopathies.

**Design, Setting, and Participants:**

This retrospective and prospective diagnostic study evaluated autopsy and biopsy skin samples from neuropathologically and clinically diagnosed patients with PD and controls without PD. Autopsy skin samples were obtained at 3 medical centers from August 2016 to September 2019, and biopsy samples were collected from 3 institutions from August 2018 to November 2019. Based on neuropathological and clinical diagnoses, 57 cadavers with synucleinopathies and 73 cadavers with nonsynucleinopathies as well as 20 living patients with PD and 21 living controls without PD were included. Specifically, cadavers and participants had PD, LBD, MSA, Alzheimer disease, progressive supranuclear palsy, or corticobasal degeneration or were nonneurodegenerative controls (NNCs). A total of 8 approached biopsy participants either refused to participate in or were excluded from this study due to uncertain clinical diagnosis. Data were analyzed from September 2019 to April 2020.

**Main Outcomes and Measures:**

Skin αSyn^P^ seeding activity was analyzed by RT-QuIC and PMCA assays.

**Results:**

A total of 160 autopsied skin specimens from 140 cadavers (85 male cadavers [60.7%]; mean [SD] age at death, 76.8 [10.1] years) and 41 antemortem skin biopsies (27 male participants [66%]; mean [SD] age at time of biopsy, 65.3 [9.2] years) were analyzed. RT-QuIC analysis of αSyn^P^ seeding activity in autopsy abdominal skin samples from 47 PD cadavers and 43 NNCs revealed 94% sensitivity (95% CI, 85-99) and 98% specificity (95% CI, 89-100). As groups, RT-QuIC also yielded 93% sensitivity (95% CI, 85-97) and 93% specificity (95% CI, 83-97) among 57 cadavers with synucleinopathies (PD, LBD, and MSA) and 73 cadavers without synucleinopathies (Alzheimer disease, progressive supranuclear palsy, corticobasal degeneration, and NNCs). PMCA showed 82% sensitivity (95% CI, 76-88) and 96% specificity (95% CI, 85-100) with autopsy abdominal skin samples from PD cadavers. From posterior cervical and leg skin biopsy tissues from patients with PD and controls without PD, the sensitivity and specificity were 95% (95% CI, 77-100) and 100% (95% CI, 84-100), respectively, for RT-QuIC and 80% (95% CI, 49-96) and 90% (95% CI, 60-100) for PMCA.

**Conclusions and Relevance:**

This study provides proof-of-concept that skin αSyn^P^ seeding activity may serve as a novel biomarker for antemortem diagnoses of PD and other synucleinopathies.

## Key Points

**Question:**

Does the pathological α-synuclein (αSyn^P^) detected by immunohistochemistry in the skin of individuals with Parkinson disease (PD) have aggregation seeding activity, and is skin αSyn^P^ seeding activity a potential biomarker for diagnosis of PD and other synucleinopathies?

**Findings:**

In this diagnostic study including skin samples from 160 autopsies and 41 biopsies, a statistically significant increase in αSyn^P^ seeding activity was observed in individuals with PD and synucleinopathies compared with controls with tauopathies and nonneurodegenerative diseases.

**Meaning:**

Skin αSyn^P^ has aggregation seeding activity in patients with PD and non-PD synucleinopathies and may be a biomarker for antemortem diagnosis of PD and other synucleinopathies.

## Teaser

This diagnostic study investigates sensitive and specific skin biomarkers for antemortem diagnosis of Parkinson disease and other synucleinopathies.

## Introduction

Parkinson disease (PD) along with other non-PD synucleinopathies, such as Lewy body dementia (LBD) and multiple system atrophy (MSA), are characterized by the accumulation of pathological α-synuclein (αSyn^P^) aggregates in the brain.^1,2^ Currently, a definite diagnosis relies on the detection of αSyn^P^-containing Lewy bodies in the brain in patients with PD and LBD or oligodendroglial cytoplasmic inclusions in patients with MSA.^3,4,5^ Development of a reliable and sensitive assay for αSyn^P^ in easily accessible peripheral tissue specimens is critical for early or differential diagnosis, determination of disease severity, and evaluation of therapeutic efficacy in clinical trials.^6^

Deposition of immunoreactive αSyn^P^ in the peripheral nervous system has been detected by immunohistochemistry (IHC) in multiple peripheral organs and tissues of patients with PD, such as the colon, salivary glands, and skin.^7,8,9,10,11^ These peripheral morphological changes have been postulated to precede brain pathology and may contribute to autonomic dysfunction, constipation, and other nonmotor symptoms in PD.^12,13,14^ Notably, phosphorylated αSyn^P^ depositions within autonomic nerve endings of biopsied skin samples have also been detected by IHC and/or immunofluorescence (IF) microscopy in living patients with PD.^13,14,15,16,17,18^ Because autonomic dysfunction associated with skin αSyn^P^ deposition may appear long before clinical symptoms in living patients with PD or individuals at prodromal stages of PD,^13,14,19^ skin αSyn^P^ is a good candidate for early diagnosis. However, detection of the phosphorylated form of skin αSyn^P^ by IHC has been challenging and inconsistent, with sensitivities ranging from 0% to 100%, possibly due to methodological variability.^8,14,20,21^ Remarkably, several recent studies have revealed that misfolded αSyn^P^, either in the brain of individuals with PD or formed in vitro by recombinant αSyn, possesses prionlike aggregation seeding activity.^22,23,24^ Nevertheless, whether skin αSyn^P^ detected by IHC and/or IF microscopy with specific antiphosphorylation antibodies has aggregation seeding activity and whether skin αSyn^P^ seeding activity, if present, can be a biomarker for diagnosis of PD and other synucleinopathies have never been determined, to our knowledge.

The seeding activity of several nonprion protein aggregates, including misfolded αSyn^P^, has been shown by 2 advanced in vitro amplification assays. One is real-time quaking-induced conversion (RT-QuIC), which was originally developed as a specific and quantitative diagnostic test for prion diseases using cerebrospinal fluid (CSF)^25,26^ and, more recently, skin tissue.^27,28^ It was recently adapted to detect αSyn^P^ seeding activity in the brain and/or CSF samples from individuals with PD and LBD with 92% to 95% sensitivity and 100% specificity.^29,30,31,32,33,34^ Protein misfolding cyclic amplification (PMCA) is another assay that has been used to detect trace amounts of misfolded prion protein aggregates in skin tissues of prion-infected mice or body fluids of patients with prion diseases.^28,35,36^ A modified version of PMCA was previously shown to detect αSyn^P^ in the CSF of patients with PD.^37^ Using RT-QuIC and PMCA assays, we report the highly sensitive and specific detection of αSyn^P^ aggregation seeding activity in autopsied and biopsied skin samples from individuals with PD and other synucleinopathies.

## Methods

### Study Oversight

The study was monitored and approved by the institutional review boards of the University Hospitals Cleveland Medical Center and Banner Sun Health Research Institute and the ethics committees of the University of Würzburg and IRCCS Institute of Neurological Sciences of Bologna. The use of human tissues was authorized by the institutional review boards or ethics committees. Written informed consent was obtained from all living participants undergoing skin biopsy and from family members for skin autopsy.

### Source of Skin Samples

A total of 130 autopsy abdominal skin samples from 130 cadavers, including 47 PD cadavers; 40 cadavers with other neurodegenerative diseases, including 7 with LBD, 3 with MSA, and 30 with tauopathies, including 17 with Alzheimer disease (AD), 8 with progressive supranuclear palsy (PSP), and 5 with corticobasal degeneration (CBD); and 43 nonneurodegenerative control (NNC) cadavers, were examined in our study (Table) (eFigure 1 in the Supplement). A total of 30 autopsy scalp skin samples were also analyzed, comprising samples from 20 of the PD cadavers whose abdominal skin specimens were analyzed and 10 additional non-PD controls (Table) (eFigure 1 in the Supplement). The samples were obtained through the Arizona Study of Aging and Neurodegenerative Disorders/Brain and Body Donation Program at Banner Sun Health Research Institute,^38^ the Human Tissue Procurement Facility at Case Western Reserve University, and University Hospitals Cleveland Medical Center. The diagnoses of these cases were confirmed by neuropathological examination of autopsied brain tissues (eTable in the Supplement).

**Table.** Demographic Characteristics and Skin Pathological α-Synuclein Thioflavin T Fluorescence of Different Groups

Variable	Autopsy sample							Biopsy sample	
	PD	LBD	MSA	AD	PSP	CBD	Control	PD	Control
Age, mean (SD), y	80.1 (7.1)	78 (6.8)	64.3 (5.9)	77.7 (8.0)	82.3 (13.1)	76.4 (4.5)	71.4 (11.8)	68.3 (7.3)	62.5 (10.2)
Samples, No.	47	7	3	17	8	5	43	20	21
Male, No. (%)	34 (72)	7 (100)	0	11 (65)	5 (63)	4 (80)	20 (47)	12 (60)	14 (67)
RT-QuIC assay									
Samples analyzed, No.	47	7	3	17	8	5	43	20	21
Mean (SD), %	71.6 (23.8)	53.1 (26.1)	51.0 (36.2)	23.2 (21.9)	17.8 (6.1)	13.3 (4.9)	12.4 (6.9)	56.6 (19.2)	8.3 (4.4)
*P* value (compared with control)^a^	<.001	<.001	.01	.47	.98	>.99	NA	<.001^b^	NA
*P* value (compared with PD)	NA	.19	.52	<.001	<.001	<.001	<.001	NA	<.001^b^
PMCA assay									
Samples analyzed, No.	24	5	3	0	5	5	8	10	10
Mean (SD), %	48.2 (22.2)	51.6 (18.0)	54.6 (44.2)	NA	26.2 (6.7)	19.5 (7.9)	11.9 (6.8)	52.2 (24.4)	24.7 (4.3)
* P* value (compared with control)^a^	<.001	.01	.03	NA	.80	.98	NA	<.005^b^	NA
* P* value (compared with PD)	NA	>.99	>.99	NA	.23	.06	<.001	NA	<.005^b^

Abbreviations: AD, Alzheimer disease; CBD, corticobasal degeneration; LBD, Lewy body dementia; MSA, multiple system atrophy; NA, not available; PD, Parkinson disease; PMCA, protein misfolding cyclic amplification; PSP, progressive supranuclear palsy; RT-QuIC, real-time quaking-induced conversion.

^a^ *P* value determined by 1-way analysis of variance.

^b^ *P* value determined by *t *test.

Living participants were diagnosed clinically based on the Movement Disorder Society Clinical Diagnostic Criteria for PD.^39^ Exclusion criteria included oral anticoagulation medication use, wound healing disorders, or allergic reactions against local anesthetics. Biopsy skin samples were obtained from the leg or the posterior cervical region of patients with PD (n = 20) and non-PD controls (n = 21) from 3 medical centers: IRCCS Institute of Neurological Sciences of Bologna, Bologna, Italy; the University Hospital of Würzburg, Würzburg, Germany; and the Human Tissue Procurement Facility, Case Western Reserve University, Cleveland, Ohio. Participants were selected during their diagnostic workup or therapy. In the Bologna biopsy group, although no patients refused to participate in the study, 2 to 3 individuals approached were excluded because of uncertain clinical diagnoses. In the Würzburg biopsy group, about 50% to 70% of all patients approached agreed to participate. The demographic data of all examined individuals are listed in the Table. More information about how we chose samples and what cases were used in each experiment is detailed in eFigure 1 in the Supplement.

### Skin Tissues and Preparation

Skin samples (approximately 30 to 100 mg each in weight; approximately 3 × 3-mm to 5 × 5-mm each in size) mainly contained epidermis and dermis. The autopsy process avoided possible cross-contamination between skin and brain tissues and between cadavers. Skin tissues (approximately 30 to 50 mg each) were washed 3 times in 1× Tris(hydroxymethyl)aminomethane–buffered saline and chopped into small pieces. The skin homogenates at 10% (weight/volume) were prepared in skin lysis buffer containing 2 mmol of calcium chloride and 0.25% (weight/volume) collagenase A (Roche) in Tris-buffered saline and incubated in a shaker at 37 °C for 4 hours, followed by homogenization in a Mini-Beadbeater (BioSpec; Laboratory Supply Network) for 1 minute. After sonication to disrupt remaining tissue structures, the samples were centrifuged for 5 minutes at 500*g* for collection of the supernatant fraction.

### RT-QuIC Analysis

RT-QuIC analysis was conducted as described previously,^27,28^ with minor modification. Briefly, the RT-QuIC reaction mix was composed of 40 mmol of phosphate buffer (pH 8.0), 170 mmol of sodium chloride, 0.1 mg/mL of recombinant human wild-type αSyn either synthesized in-house^40^ or purchased commercially (rPeptide) as designated in each analysis, 10 μmol of thioflavin T (ThT), and sodium dodecyl sulfate, 0.00125%. A 98-μL aliquot of the reaction mix was loaded into each well of a black 96-well plate with a clear bottom (Nunc) and seeded with 2 μL of skin homogenate (eMethods in the Supplement). After we observed a similar efficiency between 2 substrate preparations, we subsequently used the commercially purchased αSyn in our study.

### PMCA Analysis

Recombinant human wild-type αSyn was thawed and then centrifuged at 100 000*g* for 30 minutes at 4 °C to remove any aggregates that may have formed during the freeze-thaw process. Monomeric αSyn was diluted to 50 μmol in 10 mmol of Tris (pH 7.5) and 150 mmol of sodium chloride in 0.2-mL polymerase chain reaction tubes with 10 zirconium silicon beads (1-mm diameter; Next Advance). Polymerase chain reaction tubes were then placed in a Q700 sonicator (Qsonica) within an incubator. PMCA was carried out as previously described^40^ with repeated cycles of 10 seconds of sonication and 29 minutes, 50 seconds of incubation at 37 °C (eMethods in the Supplement).

### IHC and IF Microscopy Staining

Skin samples were fixed and embedded in paraffin for IHC and IF microscopy as previously described.^41^ See the eMethods in the Supplement for details.

### Statistical Analysis

Statistical analysis was conducted with GraphPad Prism version 8.4.3 (GraphPad Software). Experimental data were analyzed using the *t* test for comparing 2 groups and 1-way analysis of variance for more than 2. To compare RT-QuIC and PMCA analysis, we used Spearman rank correlation between paired values from the 2 assays. Percentage agreement was also assessed based on classification cutoffs from receiver operating characteristic (ROC) curve analysis. When computable, the McNemar test was used to assess marginal homogeneity and differences in agreement. Finally, we conducted the ROC curve analysis for a paired area under the ROC curve (AUC) to analyze the accuracy of skin RT-QuIC and PMCA assays.^42^ All tests adopt a 2-sided type I error level of .05.

## Results

A total of 160 autopsied skin specimens from 140 cadavers (85 male cadavers [60.7%]; mean [SD] age at death, 76.8 [10.1] years) and 41 antemortem skin biopsies (27 male participants [66%]; mean [SD] age at time of biopsy, 65.3 [9.2] years) were analyzed. Demographic characteristics are presented in the Table.

### Detection of αSyn^P^ in Autopsy Skin Samples of PD Cadavers by IHC and IF Microscopy Staining

**Figure 1.**
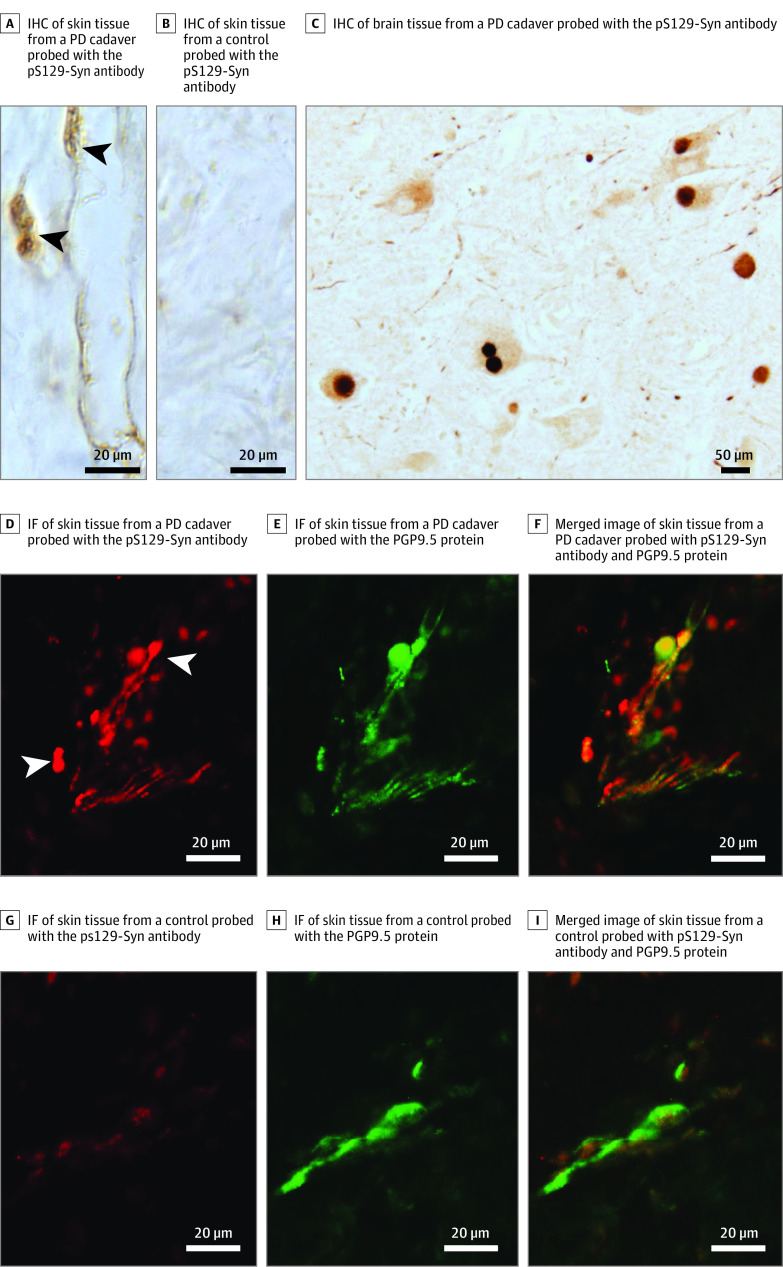
Immunohistochemistry (IHC) and Immunofluorescence (IF) Microscopy of Pathological α-Synuclein (αSyn^P^) A, IHC of skin tissue section from a Parkinson disease (PD) cadaver stained with the pS129-Syn antibody for phosphorylated αSyn^P^. B, IHC of skin tissue section from a non-PD control cadaver stained with the pS129-Syn antibody for phosphorylated αSyn^P^. C, IHC of brain tissue section from a PD cadaver stained with the pS129-Syn antibody for phosphorylated αSyn^P^. D, IF microscopy of skin tissue section from a patient with PD stained with the pS129-Syn antibody. E, IF microscopy of skin tissue section from a patient with PD stained with the PGP9.5 antibody directed against an axonal marker PGP9.5 protein. F, Merged image of IF microscopy of skin tissue section from a patient with PD stained with the pS129-Syn antibody and PGP9.5 antibody. G, IF microscopy of skin tissue section from a control without PD stained with the ps129-Syn antibody. H, IF microscopy of skin tissue section from a control without PD stained with the PGP9.5 protein. I, Merged image of IF microscopy of skin tissue section from a control without PD stained with the pS129-Syn antibody and PGP9.5 antibody.

We first used IHC and IF microscopy to reaffirm the presence of phosphorylated αSyn^P^ in the skin tissues of PD cadavers. Consistent with the previous findings,^10,11,13,14,15,16,17,18,19,20,21^ our analyses confirmed that phosphorylated αSyn^P^ was detectable with pS129-Syn (the antibody directed against phosphorylated α-synuclein) colocalized with PGP9.5 (an axonal marker), revealing the presence of phosphorylated αSyn^P^ in the skin nerve fibers of PD cadavers (Figure 1A and C) but not cadavers without PD by IHC (Figure 1B) and IF microscopy (Figure 1D-I).

### Detection of αSyn^P^ Seeding Activity in PD Autopsy Skin Samples Using RT-QuIC and PMCA Assays

**Figure 2.**
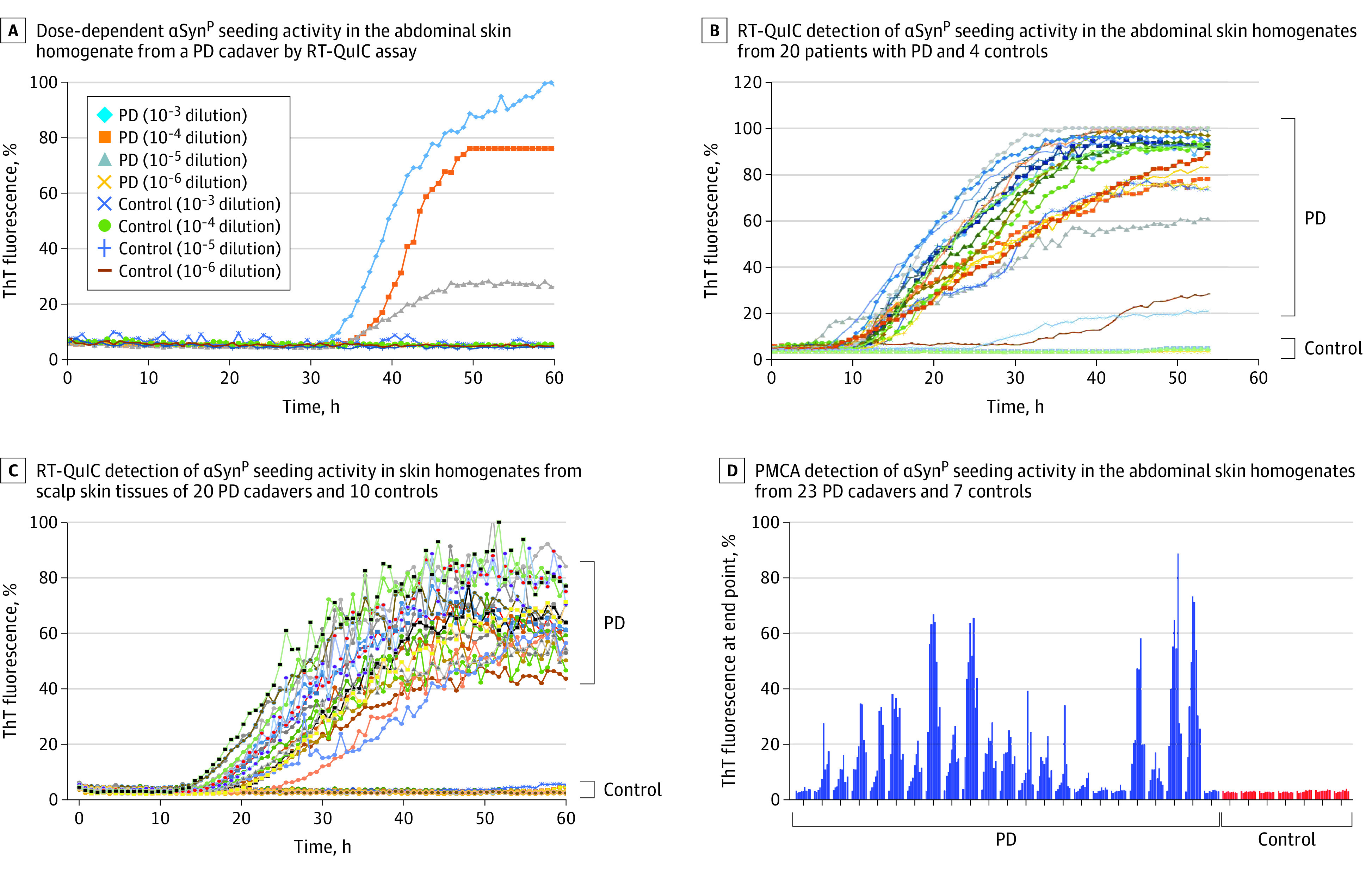
Detection of Pathological α-Synuclein (αSyn^P^) Seeding Activity in Autopsy Skin Samples From Parkinson Disease (PD) Cadavers by Real-Time Quaking-Induced Conversion (RT-QuIC) or Protein Misfolding Cyclic Amplification (PMCA) Assay A, Dose-dependent αSyn^P^ seeding activity in the abdominal skin homogenate from a PD cadaver by RT-QuIC assay. Abdominal skin homogenate from a control was used as a negative control. The skin homogenates were diluted to 10^−3^ through 10^−6^ dilution. Commercially purchased recombinant human αSyn (rPeptide) was used. B, RT-QuIC detection of αSyn^P^ seeding activity in the abdominal skin homogenates from 20 patients with PD and 4 controls with PD. Synthesized in-house recombinant human αSyn was used. C, RT-QuIC detection of αSyn^P^ seeding activity in skin homogenates from the scalp skin tissues of 20 patients with PD and 10 additional controls without PD, all of which were coded for the blinded test. Commercially purchased recombinant human αSyn (rPeptide) was used. D, PMCA detection of αSyn^P^ seeding activity in the abdominal skin homogenates from 23 patients with PD and 7 controls without PD. Synthesized in-house recombinant human αSyn was used. All data were normalized to percentages of the maximal fluorescence response.

We used RT-QuIC to determine whether skin αSyn^P^ has aggregation seeding activity. Neuropathologically confirmed PD and non-PD autopsy skin samples were serially diluted, and RT-QuIC was able to detect αSyn^P^ seeding activity in PD samples serially diluted to 10^−5^ but not at 10^−6^ dilution, whereas no seeding activity was observed at any of the 4 dilutions (10^−3^ to 10^−6^ dilution) in non-PD samples up to 60 hours (Figure 2A).

Autopsy abdominal skin samples from 20 PD and 4 non-PD cadavers were examined by RT-QuIC. The mean (SD) maximal ThT fluorescence was 82.6% (22.5) in PD cadavers, which was significantly higher than that of non-PD controls (mean [SD], 4.5% [0.7]; *P* < .001) (Figure 2B). A total of 2 of 20 PD cadaver skin samples exhibited a notably lower response, although it was still significantly higher than that of non-PD skin controls; one of them was still above the threshold that defined a positive RT-QuIC response based on the average of negative controls plus 3-fold SD. According to this definition, our RT-QuIC analysis yielded 95% sensitivity (95% CI, 85-100) and 100% specificity (95% CI, 78-100).

We next examined posterior scalp skin samples from the same 20 PD cadavers and 10 additional NNCs that were all blindly coded for unbiased detection. We obtained 100% sensitivity (95% CI, 91-100), and specificity remained at 100% (95% CI, 87-100) (Figure 2C). Together, our results indicated that αSyn^P^ seeding activity can be specifically detected in skin samples of PD cadavers, and scalp skin had a higher sensitivity compared with abdominal skin.

Using PMCA, we also detected αSyn^P^ seeding activity in autopsy skin homogenates. Of 23 PD abdominal skin samples, 19 (83%) exhibited positive αSyn^P^ seeding activity. In contrast, none of the 7 NNCs showed detectable seeding activity (Figure 2D). The PMCA analysis of these skin samples exhibited 83% sensitivity (95% CI, 74-92) and 100% specificity (95% CI, 84-100).

### Comparison of αSyn^P^ Seeding Activity in Autopsy Skin Samples Between Patients With Synucleinopathies and Nonsynucleinopathies

**Figure 3.**
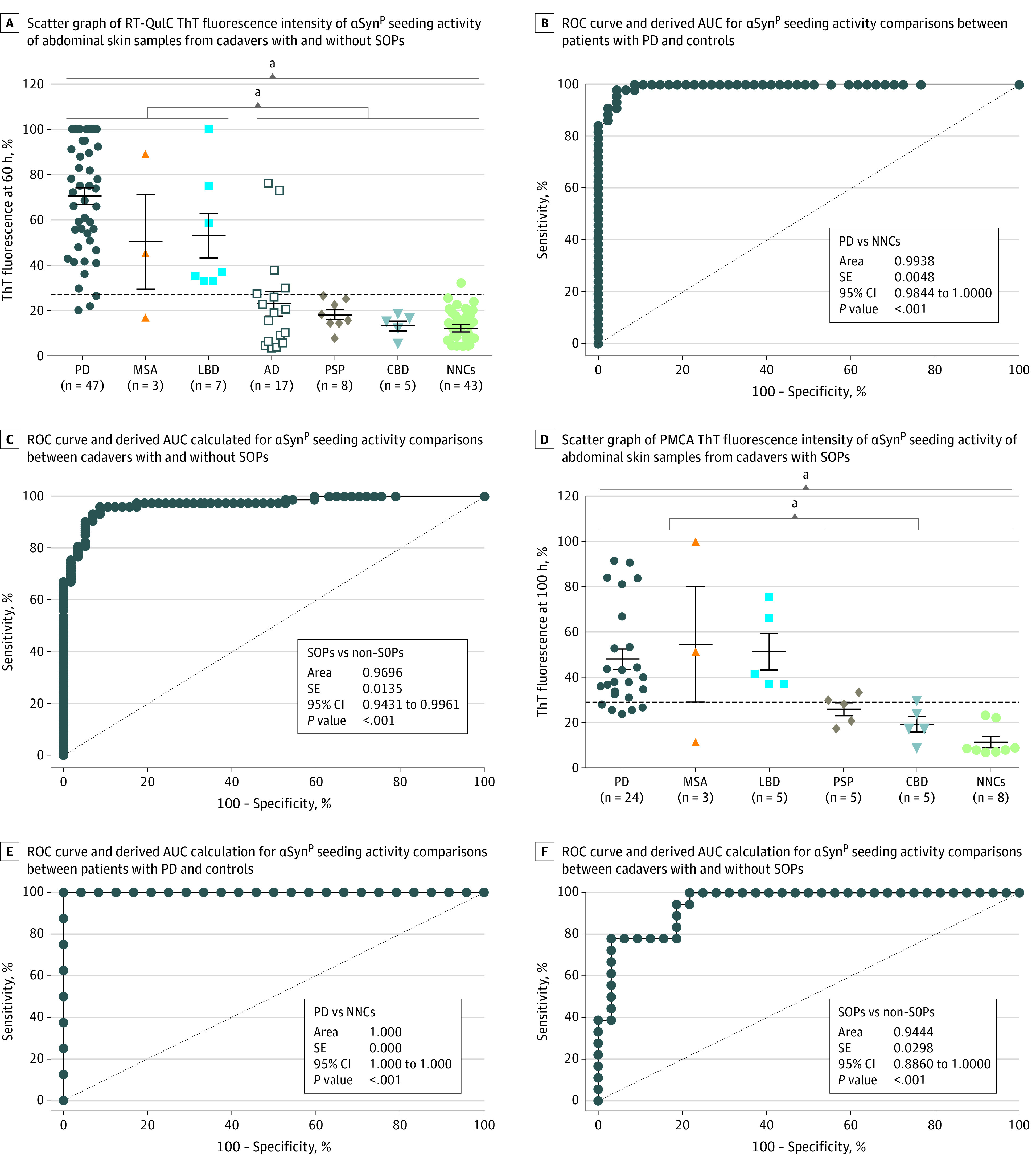
Comparison of Pathological α-Synuclein (αSyn^P^) Seeding Activity in Autopsy Skin Samples From Cadavers With Synucleinopathies (SOPs), Tauopathies, or Nonneurodegenerative Controls (NNCs) by Real-Time Quaking-Induced Conversion (RT-QuIC) and Protein Misfolding Cyclic Amplification (PMCA) Assays A, Scatter graph of RT-QuIC thioflavin T (ThT) fluorescence intensity at 60 hours of αSyn^P^ seeding activity of abdominal skin samples from cadavers with SOPs, including 47 Parkinson disease (PD) cadavers, 3 multiple system atrophy (MSA) cadavers, and 7 Lewy body dementia (LBD) cadavers, as well as non-SOPs, including 17 Alzheimer disease (AD) cadavers, 8 progressive supranuclear palsy (PSP) cadavers, 5 corticobasal degeneration (CBD) cadavers, and 43 nonneurodegenerative controls (NNCs). B and C, Receiver operating characteristic (ROC) curves and derived area under the curve (AUC) calculations for αSyn^P^ comparisons between PD and NNC cadavers and between SOP and non-SOP cadavers. D, Scatter graph of PMCA ThT fluorescence intensity at 100 hours of αSyn^P^ seeding activity of abdominal skin samples from cadavers with SOPs, including 24 PD cadavers, 3 MSA cadavers, and 5 LBD cadavers, as well as non-SOPs, including 5 PSP cadavers, 5 CBD cadavers, and 8 NNC cadavers. E and F, ROC curve and AUC calculation for comparisons of αSyn^P^ seeding activity between PD and NNC cadavers and between SOP and non-SOP cadavers. The dotted lines indicate the threshold defining the positive and negative cases. Commercially purchased recombinant human αSyn was used. ^a^*P* < .001.

To validate our findings, more autopsy abdominal skin samples from additional neuropathologically confirmed individuals with PD (n = 27) and NNCs (n = 39) were examined. We also included other synucleinopathies (LBD and MSA) and tauopathies (AD, PSP, and CBD) (Table) (eFigure 1 in the Supplement). Positive αSyn^P^ seeding activity was present in skin samples from 44 of 47 PD cadavers (including 27 new cases), 2 of 3 MSA cadavers, and all 7 LBD cadavers, which yielded 94% sensitivity (95% CI, 81-98) for PD, 67% sensitivity (95% CI, 42-92) for MSA, 100% sensitivity (95% CI, 94-100) for LBD, and 93% sensitivity (95% CI, 85-97) for all 3 synucleinopathies combined (Figure 3A). In contrast, αSyn^P^ seeding activity was only observed in 1 of 43 NNCs and 5 of 17 AD skin samples (Figure 3A). None of the 5 CBD and 8 PSP skin samples showed positive seeding activity. As a result, the specificity of RT-QuIC was 93% (95% CI, 83-97) when PSP, CBD, AD, and NNCs were combined as a group of nonsynucleinopathies. The specificity was 98% (95% CI, 89-100) if compared only with NNCs (Figure 3A). ROC curve analysis confirmed that the skin RT-QuIC maximum relative ThT fluorescence responses were highly accurate in discriminating PD (n = 47) from NNCs (n = 43) (mean [SE] AUC, 0.9938 [0.0048]; 95% CI, 0.9844-1.0000; *P* < .001) (Figure 3B). The skin RT-QuIC assay was also able to highly accurately differentiate synucleinopathies (n = 57) from nonsynucleinopathies (n = 73) (mean [SE] AUC, 0.9696 [0.0135]; 95% CI, 0.9431-0.9961; *P* < .001) (Figure 3C) (Table). More detailed demographic characteristics and comparisons of αSyn^P^ seeding activity among different groups are listed in the Table.

Some of the above autopsy skin samples from PD cadavers (n = 24), LBD cadavers (n = 5), MSA cadavers (n = 3), PSP cadavers (n = 5), and CBD cadavers (n = 5) and NNCs (n = 8) were also examined by PMCA. Consistent with RT-QuIC findings, PMCA maximum relative ThT fluorescence responses from skin samples were highly accurate in discriminating synucleinopathies (n = 32) from nonsynucleinopathies (n = 18) (mean [SE] AUC, 0.9444 [0.0298]; 95% CI, 0.8860-1.0000; *P* < .001) (Figure 3B) (Table). Combining this set of data and the one shown in Figure 2D, the sensitivity and specificity of PMCA to differentiate synucleinopathies from nonsynucleinopathies were 82% (95% CI, 76-88) and 96% (95% CI, 85-100), respectively.

To analyze the agreement levels of RT-QuIC and PMCA data for αSyn^P^ seeding activity in the cadavers who were examined by both assays, we conducted stratified analyses using Spearman rank correlations by each disease (PD [n = 24]: *r* = 0.44; *P* = .03; MSA [n = 3]: *r* = 0.50; *P* > .99; LBD [n = 5]: *r* = 0.90; *P* = .04; PSP [n = 5]: *r* = 0.80; *P* = .10; CBD [n = 5]: *r* = 0.80; *P* = .10) (eFigure 2 in the Supplement). In terms of percentage agreement using RT-QuIC and PMCA cutoffs, PD agreement percentage was 78.6% (95% CI, 62.2-95.0), with RT-QuIC having slightly higher observed sensitivity than PMCA. Percentage agreement for the other diagnosis groups was 100%. For both methods, the MSA group had 1 false-negative out of 3 cases, whereas the rest were 100% accurate. Among PD cases only, there was 75% agreement, and the McNemar test indicated no significant difference (*P* = .69) (eFigure 2 in the Supplement), which also supports agreement between methods. For MSA, findings from the McNemar test were nonsignificant; it could not be computed for the other diagnostic groups since both approaches included samples that were either all positive or negative. ROC analysis of PD only vs CBD and PSP resulted in AUC values for RT-QuIC and PMCA of 0.975 (95% CI, 0.931-1.000) and 0.913 (95% CI, 0.816-1.000), respectively. No significant difference when comparing AUC values was observed (*P* = .27).^42^ All these statistical analyses suggested that the 2 assays were significantly consistent in detecting skin αSyn^P^ seeding activity, mirroring their success in detecting skin prion seeding activity in prion disease.^28^

### Detection of αSyn^P^ Seeding Activity in Skin Biopsy Samples From Living Patients With PD

**Figure 4.**
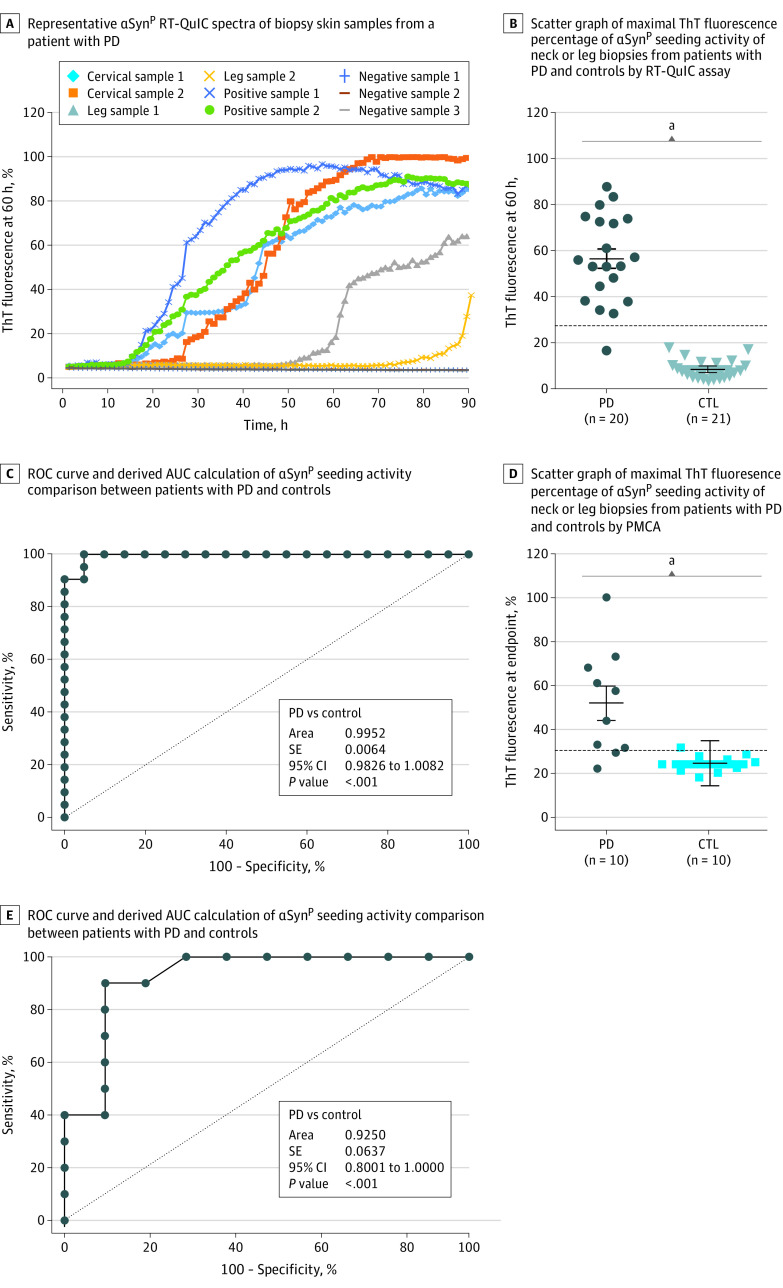
Detection of Pathological α-Synuclein (αSyn^P^) Seeding Activity in Biopsy Skin Samples From Living Patients With Parkinson Disease (PD) and Controls Without PD by Real-Time Quaking-Induced Conversion (RT-QuIC) and Protein Misfolding Cyclic Amplification (PMCA) Assays A, Representative αSyn^P^ RT-QuIC spectra of biopsy skin samples from 2 posterior neck areas (cervical samples 1 and 2) and 2 leg areas (leg samples 1 and 2) of a living patient with PD. Positive autopsy skin controls from PD cadavers (positive samples 1 and 2) and negative autopsy controls from non-PD cadavers (negative samples 1, 2, and 3) were included. Data were normalized to percentage of the maximal fluorescence response. B, Scatter graph of maximal thioflavin T (ThT) fluorescence percentage at 60 hours of the αSyn^P^ seeding activity of the posterior neck or leg biopsy skin samples from 20 living patients with PD and 21 living controls without PD by RT-QuIC assay. C, ROC curve and AUC calculation for comparison of αSyn^P^ seeding activity between patients with PD and controls without PD. D, Scatter graph of maximal ThT fluorescence percentage at 100 hours of αSyn^P^ seeding activity of the posterior neck or leg biopsy skin samples from 10 living patients with PD and 10 living controls without PD by PMCA. E, ROC curve and AUC calculation between PD and non-PD controls. The dotted lines represent the thresholds defining the samples with positive vs negative findings. Commercially purchased recombinant human αSyn was used. ^a^*P* < .001.

To determine whether the above findings can be applied to skin biopsy samples from living patients with PD, skin samples obtained by punch biopsy from 2 posterior cervical sites (C7) and 2 leg sites were examined by RT-QuIC. Like the 2 positive control PD cadaver skin samples, the 2 posterior cervical skin biopsy samples showed positive seeding activity (Figure 4A). In contrast, the 2 leg skin biopsy samples exhibited weak seeding activity only after 50 or 70 hours. No αSyn^P^ seeding activity was detected in the 3 negative skin specimens from non-PD controls (Figure 4A).

We next tested biopsy samples from living patients with PD (n = 20) and non-PD living controls (n = 21) using the RT-QuIC assay. The maximum responses in the biopsy skin samples were significantly higher in patients with PD than in non-PD controls (mean [SD] response, 56.6% [19.2] vs 8.3% [4.4]; *P* < .001) (Figure 4B) (Table). ROC analysis showed that living patients with PD were highly accurately differentiated from non-PD controls by our skin-based assay (mean [SE] AUC, 0.9952 [0.0064]; 95% CI, 0.9826-1.0082; *P* < .001) (Figure 4B) (Table). The RT-QuIC assay revealed 95% sensitivity (95% CI, 77-100) and 100% specificity (95% CI, 84-100). PMCA also accurately differentiated patients with PD (n = 10) from non-PD controls (n = 10) at the end point of 100 hours (mean [SE] AUC, 0.9250 [0.0637]; 95% CI, 0.8001-1.0000; *P* < .001) (Figure 4C) (Table) with 80% sensitivity (95% CI, 49-96) and 90% specificity (95% CI, 60-100).

## Discussion

The aggregation seeding capability of αSyn^P^ led to the development of RT-QuIC and PMCA platforms to sensitively detect αSyn^P^. These assays have recently been applied to CSF and autopsied submandibular gland tissues.^29,30,31,32,33,34,37,43^ However, because of the invasive nature of lumbar puncture for CSF collection, it is not practical to collect CSF for routine early detection of αSyn^P^ during clinical visits or for serial evaluation during clinical trials. Moreover, lumbar puncture for CSF sampling is not always feasible because of relative contraindications, such as anticoagulation, particularly common in elderly patients. In addition, submandibular gland biopsy revealed significantly higher adverse events than skin biopsy based on a recent study that evaluated feasibility and safety of multicenter tissue and biofluid sampling for detection of αSyn^P^ in patients with PD.^44^

To our knowledge, this study has demonstrated for the first time that skin αSyn^P^ has aggregation seeding activity that was significantly higher in individuals with PD and other synucleinopathies than in those with tauopathies and NNCs. The skin-based analyses provided comparable diagnostic sensitivity and specificity to CSF-based assays. Given that skin punch biopsy is relatively easier to perform and much less invasive, skin αSyn^P^ seeding activity may be a more practical antemortem diagnostic biomarker for PD and synucleinopathies. Further studies will be needed to determine if αSyn^P^ seeding activity can be detected in skin preclinically in these diseases. This possibility is supported by our recent findings that skin prion seeding activity is detectable not only in all patients with Creutzfeldt-Jakob disease^27^ but also far in advance of neuronal damage and clinical signs of prion disease in rodents.^28^

To our knowledge, there is currently no antemortem diagnostic test to reliably differentiate tauopathies from synucleinopathies in living patients with parkinsonism. Such a test would aide in treatment planning, prognostication, and guiding enrollment in clinical studies. Here, we showed that RT-QuIC and PMCA analyses of skin αSyn^P^ seeding activity among patients with parkinsonism can differentiate synucleinopathies (PD, MSA, and LBD) from tauopathies (PSP and CBD), which is consistent with previous reports of detecting αSyn^P^ deposits in the skin nerve fibers of patients with LBD and MSA with IHC or IF microscopy.^16,45,46^ Since the cases of non-PD synucleinopathies and tauopathies examined in this study were much fewer than PD cases, more studies are needed to validate this finding.

Positive αSyn^P^ seeding activity was observed in autopsy skin specimens from 5 of 17 AD cadavers by RT-QuIC and 1 of 5 PSP cadavers by PMCA, which resulted in a decreased detection specificity. Notably, the coexistence of different misfolded proteins has been reported to occur in individual patients; specifically, some brains with neuropathologically confirmed AD, PSP, and CBD were reported to contain Lewy bodies concurrent with deposits of β-amyloid and/or tau.^47,48,49,50,51^ Thus, it would be important to examine both the brain and skin tissues of these individuals to determine whether the 2 types of tissues are consistent in having αSyn^P^ seeding activity.^52^

We found that the sensitivity and specificity of RT-QuIC were higher in posterior scalp than in abdominal skin tissues. Moreover, the skin biopsy tissues from the posterior cervical region (lateral to the C7 vertebra) of patients with PD exhibited higher and earlier αSyn^P^ seeding activity compared with the leg skin tissues. Thus, standardizing RT-QuIC with posterior cervical skin may ultimately lead to a quantitative assay to measure skin αSyn^P^ seeding activity at different stages of synucleinopathies, which will be a great complement to imaging tests to monitor disease progression. Moreover, our RT-QuIC and PMCA assays can also complement the ongoing investigations of peripheral PD biomarkers using IHC detection of αSyn^P^,^14,19^ which will allow us to correlate the biological activity to the morphology of αSyn^P^ aggregates in different skin areas.

### Limitations

This study has limitations. For instance, compared with PD and non-PD controls, the number of cases with non-PD synucleinopathies, such as MSA and LBD, or tauopathies, such as PSP and CBD, is relatively small. Moreover, the number of skin biopsy samples is also limited. Our findings need to be further validated with a large number of cases. Another limitation of our study is that the sensitivity and specificity of RT-QuIC and PMCA assays for αSyn^P^ seeding activity in the biopsy skin samples from living patients were mainly based on the clinical diagnoses of PD and non-PD synucleinopathies. They could be highly variable, mainly dependent on how accurate the clinical diagnosis of individuals in each cohort evaluated is. A follow-up neuropathological autopsy of each case will be critical to validate the sensitivity and specificity of the biopsy skin samples.

## Conclusions

Using RT-QuIC and PMCA assays, we provide the first evidence that skin αSyn^P^ has aggregation seeding activity. Our findings suggest that skin αSyn^P^ seeding activity is a feasible biomarker for diagnosis of PD and possibly other synucleinopathies.

## Notes

Supplement. **eMethods.** **eFigure 1.** Flowchart of cases examined in each experiment. **eFigure 2.** Statistical comparison of RT-QuIC and PMCA assays. **eTable.** Neuropathological features of cases in different groups examined.
