# Supplementary material for: Skin α-Synuclein Aggregation Seeding Activity as a Novel Biomarker for Parkinson Disease
Source: JAMA Neurol. 2020 Sep 28;78(1):1–11. doi: 10.1001/jamaneurol.2020.3311 (PMC7522783; doi:10.1001/jamaneurol.2020.3311)
Supplement: Supplement. — eMethods. eFigure 1. Flowchart of cases examined in each experiment. eFigure 2. Statistical comparison of RT-QuIC and PMCA assays. eTable. Neuropathological features of cases in different groups examined. [file jamaneurol-e203311-s001.pdf]

Supplementary Online Content

Wang Z, Becker K, Donadio V, et al. Skin α-synuclein aggregation seeding activity as a novel biomarker for Parkinson disease. *JAMA Neurol*. Published online September 28, 2020. doi:10.1001/jamaneurol.2020.3311

eMethods.

eFigure 1. Flowchart of cases examined in each experiment

eFigure 2. Statistical comparison of RT-QuIC and PMCA assays

eTable. Neuropathological features of cases in different groups examined

This supplementary material has been provided by the authors to give readers additional information about their work.

© 2020 Wang Z et al. *JAMA Neurology.*

eMethods.

Source of Skin Samples

Based mainly on their availability, samples were randomly chosen for each experiment. Specifically, we started with 20 PD and 4 non-PD autopsy abdominal skin samples available to set up the RT-QuIC and PMCA assay conditions and then blindly tested scalp skin samples from the same 20 PD while they were re-coded by the sample provider along with 10 additional coded non-PD samples. We subsequently validated our findings with an increased number of PD and control cases including other synucleinopathies and tauopathies. Finally, we tested our assays with all biopsy samples available.

RT-QuIC Analysis

The skin homogenates were spun at 2,000 g for 2 min at 4°C prior to making serial dilutions. The 96-well plate was then sealed with a plate sealer film (Nalgene Nunc International) and incubated at 42°C in a BMG FLUOstar Omega plate reader with cycles of 1 min shaking (400 rpm double orbital) and 1 min rest throughout the indicated incubation time. ThT fluorescence measurements (450 +/-10 nm excitation and 480 +/-10 nm emission; bottom read) were taken every 45 minutes. Four replicate reactions were seeded with the same dilution of an individual sample. The average fluorescence values per sample were calculated using fluorescence values from all four replicate wells regardless of whether these values crossed the threshold described below. To compensate for minor differences in baselines between fluorescent plate readers and across multiple experiments, some data sets were normalized to a percentage of the maximal fluorescence response (260,000 rfu) of the plate readers as described,[27,28] and plotted versus different reaction times. Reactions were classified as RT-QuIC positive based on criteria similar to those previously described. [27,28] A ThT fluorescence threshold for a reaction to be considered

© 2020 Wang Z et al. *JAMA Neurology.*

positive was based on the mean ThT value of all negative control samples at 60 hours, plus 3 standard deviations yielding an ~27% threshold. At least 2 of 4 replicate wells must cross this threshold and lag phase less than 50 hours for a sample to be considered positive.

PMCA Analysis

At various time points, a 2 µL of sample aliquot was removed and incubated with 198 µL ThT solution (20 µM ThT, 50 mM glycine, pH 8.5) in a 96-well plate (Greiner Bio-One) for 5 minutes at room temperature. ThT readings were performed on a BioTek plate reader with an optimized gain of 100 (excitation at 440 nm and emission at 480 nm).

Immunohistochemistry and Immunofluorescence staining

Immunohistochemistry and immunofluorescence staining were conducted as previously described.[41] Antigen retrieval with pressure cooking using decloaking buffer (Biocare) or with 80% formic acid for 30 min was performed prior to blocking with 10% normal goat serum (NGS). Primary antibodies (PGP9.5, Genetex; and alpha synuclein Ser 129, BioLegend) were incubated overnight. For immunohistochemical staining, goat-anti-mouse or goat-anti-rabbit secondary antibodies (Millipore) were incubated for 30 minutes followed by a 60 min incubation in either mouse or rabbit peroxidase-anti-peroxidase (Jackson ImmunoResearch). After rinsing in Tis buffer (50 mM Tris, pH = 7.6) sections were developed with DAB reagent (Biocare), dehydrated and cover-slipped. For double label immunofluorescence, sections were rehydrated and primary antibodies were incubated together overnight. After rinsing in PBS, Alexafluor 488 and 568 species specific conjugated secondary antibodies (Invitrogen) were incubated at RT for 1 hr. After 3 rinses in PBS, sections were subjected to autofluorescence quenching step (Vector TrueVIEW kit), and cover slipped with Vectashield with DAPI mounting media. Images were obtained on a Zeiss Axiovert.

© 2020 Wang Z et al. *JAMA Neurology.*

eFigure 1. Flowchart of cases examined in each experiment

A1 through A140 represent individual cadavers with autopsy skin samples examined by RT-QuIC and/or PMCA assays. B1 through B41 represent individual living subjects with biopsy skin samples examined by RT-QuIC and/or PMCA assays.

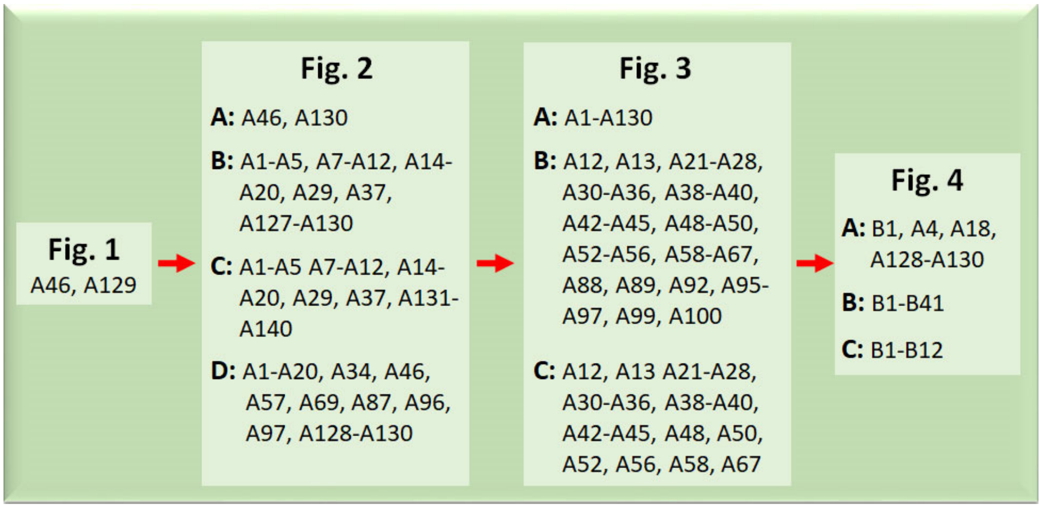

© 2020 Wang Z et al. *JAMA Neurology.*

eFigure 2. Statistical comparison of RT-QuIC and PMCA assays

(***A***) Bar graph of positive and negative PD cases detected by RT-QuIC and PMCA assays of αSyn[P] seeding activity in autopsy abdominal skin samples from 24 PD cadavers. *: Spearman rank correlation r = 0.68 (p =.027 < .05) between RT-QuIC and PMCA. (***B***) Agreement level of RT-QuIC and PMCA assays of αSyn[P] seeding activity. PD agreement percentage between RT-QuIC and PMCA was 78.6% with McNemar's test p = .69 >.05, which supports agreement between the two assays. (***C***) A paired AUC analysis of PD only versus CBD and PSP. AUC values for RT-QuIC and PMCA respectively were 0.94 and 0.96 (p = 0.66 >.05), suggesting that the two methods were highly accurate and were comparable.

A

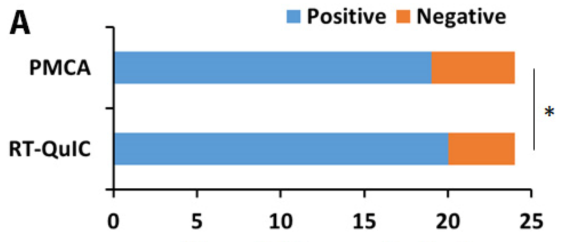

B

Agreement table		
PD (n=24)	PMCA Negative	PMCA Positive
RT-QuIC Negative	2	2
RT-QuIC Positive	4	16

C

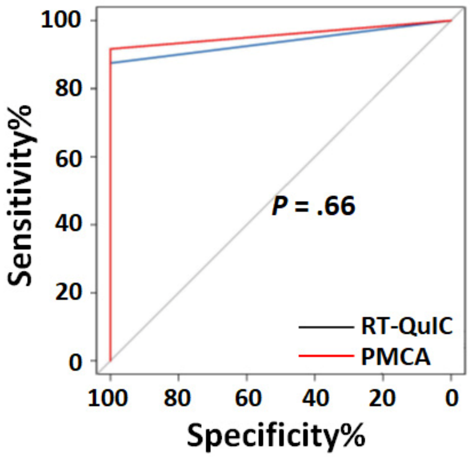

© 2020 Wang Z et al. *JAMA Neurology.*

eTable. Neuropathological features of cases in different groups examined

Neuropathological Diagnosis	No. Case	Neuropathological Features
**Parkinson's Disease (PD)**	**47**	36% Braak stage III brainstem/limbic changes and 64% Braak stage IV neocortical changes. 34% had argyrophilic grains (AG), 34% microscopic changes of AD but insufficient for diagnosis (MCADID), 23% neurofibrillary tangles (NFTs), 19% concomitant AD pathology, 9% with PSP and 4% with CBD pathological changes, and 4% with cerebral amyloid angiopathy (CAA).
**Lewy Body Dementia (LBD)**	**7**	29% cases with CBD, 29% with AG, 14% with AD, 14% hippocampal sclerosis with phosphorylated TDP-43, and 14% MCADID.
**Multiple System Atrophy (MSA)**	**3**	MSA-related fndings including αSyn-positive mainly in Glial cytoplasmic inclusions (GCIs), and 33% cases with NFTs.
**Alzheimer's Disease (AD)**	**17**	Intermediate level AD neuropathological changes, neurofibrillary degeneration (Braak Stage I-IV), 24% CAA, and 6% AG.
**Progressive Supranuclear Palsy (PSP)**	**8**	NFTs in subcortical and brainstem regions, 75% with MCADID, and 63% with AG.
**Corticobasal Degeneration CBD)**	**5**	Tau pathology in the primary motor and somatosensory cortices and putamen, 80% cases with AG, 40% with AD, 20% with PD, and 40% microscopic infarct.
**Non-neurodegenerative Disease Controls**	**43**	37% cases with no detectable neuropathology, 35% various infarcts including lacunar-sized acute or subacute infarct, cavitated or organzing infarct, 12% MCADID, 12% atherosclerosis, 2% NFTs, and 2% CAA.

© 2020 Wang Z et al. *JAMA Neurology.*
